# Investigation of Geriatric Patients with Abdominal Pain Admitted to Emergency Department

**DOI:** 10.1155/2018/9109326

**Published:** 2018-05-30

**Authors:** Pınar Henden Çam, Ahmet Baydin, Savaş Yürüker, Ali Kemal Erenler, Erdinç Şengüldür

**Affiliations:** ^1^Ondokuz Mayıs University, Department of Emergency Medicine, Samsun, Turkey; ^2^Ondokuz Mayıs University, Department of General Surgery, Samsun, Turkey; ^3^Hitit University, Department of Emergency Medicine, Çorum, Turkey

## Abstract

**Introduction:**

The aim of this study is to detect the possible reasons of abdominal pain in the patients aged 65 and older admitted to emergency department (ED) with complaint of abdominal pain which is not related to trauma, to determine the length of hospitalization of old (65–75 age) and elderly (aged 75 and older) patients, and to define the hospitalization and mortality rates.

**Material and Methods:**

In the study, 336 patients were included. Groups were compared in respect to gender, internal or surgical prediagnoses, complaints accompanying abdominal pain, vital findings, comorbidities, requested consultations, hospitalizing service, waiting time in the ED and in the hospital, and treatment methods.

**Results:**

Of the patients, 48.2% were male, and 51.8% were female. While 52.4% of the patients were in 65–74 age group, 47.6% of them were aged 75 years and above. An internal disease was detected in 76.8% of the patients as an origin of abdominal pain. Most common prediagnoses were biliary diseases and diseases related to biliary tract followed by nonspecific abdominal pain, abdominal pain secondary to malignity, ileus, and acute gastroenteritis, respectively. The most frequent finding accompanying abdominal pain was vomiting. The most frequent chronic disease accompanying abdominal pain was hypertension in both age groups. We observed that 75.9% of the patients required consultation. We detected that 48.8% of the patients with abdominal pain were hospitalized and they were hospitalized mostly by gastroenterology ward (24.8%). Surgical treatments were applied to the 17.6% of the patients with abdominal pain.

**Conclusion:**

Clinical findings become indistinct by age, and differential diagnosis of abdominal pain gets more difficult in geriatric patients. Therefore, physicians should consider age related physiological changes in order to distinguish geriatric patients admitted to emergency service with abdominal pain from pathological cases requiring immediate surgical operation.

## 1. Introduction

The number of elderly people (≥65 years old) is increasing both in Turkey and internationally due to improved living conditions and decreased mortality rates. Knowing the characteristics of elderly patients admitted to emergency departments can provide guidance for diagnosis and treatment approaches [1]. As the number of elderly patients increases, the number of elderly patients admitted to the EDs increases. Various studies have shown that percentage of geriatric patients admitted to EDs vary between 9 and 19% and these patients are known to present with more severe clinical situation when compared to younger patients [2–5]. While abdominal pain is about 10% of all complaints presenting to EDs, 20% of these patients are known to be geriatric patients. More than half of these patients are being hospitalized and surgical intervention is performed to 1/3 of the patients. Fagbohun et al. reported that biliary system diseases are the most common source of abdominal pain and the primary reason for surgery [6]. Mortality rate for patients older than 65 years has been reported to vary between 11 and 14%. Mc Namara et al. reported that reasons for high mortality rate in geriatric patients were related to comorbidities, former surgical procedures, multiple drug use, impotent immune system, and delayed recognition of serious conditions in the ED [7]. Despite improvements in management of geriatric patients in the ED, geriatric patients remain to be a clinical challenge for ED physicians. In this study, our aim was to investigate reasons, prevalence, hospitalization rates, demographical features, and morbidity and mortality rates of patients older than 65 years admitted to our ED due to abdominal pain and guide physicians and hospital managers in geriatric patient care.

## 2. Material and Methods

After ethical approval, medical records of patients older than 65 years admitted to our ED with abdominal pain in the last year were investigated retrospectively. Inclusion criteria were patients with abdominal pain older than 65 years. Medical records of the patients were detected for age, gender, vital signs, complaints accompanying abdominal pain (nausea-vomiting, loss of appetite, constipation, intestinal gas extraction inability, diarrhea, dysuria, jaundice), comorbidities, duration of abdominal pain, length of ED stay (0–6, 6–12, 12–24 and over 24 hours), prediagnosis in the ED, consultations required (internal medicine, general surgery, urology, gynecology, etc.), wards that the patients were admitted to (ED, internal medicine, general surgery, intensive care, etc.), length of stay (LOS) of the patients in the hospital (1 day, 1–4 days, 4–10 days, more than 10 days), choice of treatment (medical, surgical), and outcomes (recovery, vegetative stage, exitus). The patients were divided into subgroups according to age (65–74 years and above 75 years) and diagnoses (medical and surgical). Patients under 65 years and with traumatic abdominal pain were excluded from the study.

Data were analyzed using Statistical Package for Social Sciences (SPSS) for Windows® 21.0 programme. Frequency (*n*) and percentage (%) were given for categorical variables. Median, minimum, and maximum values were given for continuous variables. In comparison of categorical variables, Pearson's chi-square test and Student *T*-test were used. For comparison of body temperature between patient groups, Mann–Whitney *U* test was used. *p* < 0.05 was considered as statistically significant.

## 3. Results

Into the study, 336 patients over 65 years with abdominal pain were included between the study periods. Of the patients, 162 were male (48.2%) and 174 were female (51.8%). While 176 patients were involved in 65–74 years group (52.4%), 160 were involved in 75 years and above group (47.6%).

Mean age of the patients was 74.8 ± 6.5 years (min: 65 years, max: 96 years). While mean age of female patients was 75.3 ± 6.0, mean age of male patients was 74.2 ± 6.0. Mean age of the patients according to gender was not statistically significant (*p* > 0.05). Comparison of patient characteristics according to age groups is summarized in Table 1.

When source of abdominal pain was investigated, 258 patients had a medical source (76.8%) and 78 had a surgical source (23.2%). Of the patients with medical diagnoses, 53.5% were in 65–74 years group and 46.5% were in 75 years and above group. Of the patients with surgical diagnoses, 48.7% were in 65–74 years group and 51.3% were in 75 years and above group. No statistical significance could be determined when patients were investigated according to age, gender, and source of abdominal pain (*p* > 0.05). When complaints accompanying abdominal pain were compared between age groups, no statistical significance could be determined.

When complaints accompanying abdominal pain were compared, nausea/vomiting and gas extraction inability were found to be statistically significant in males (*p* < 0.05).

Additionally, when complaints were compared as medical and surgical sources, diarrhea, jaundice in medical sources, and gas extraction inability in surgical sources were found significantly higher (*p* > 0.05).  Table 2 summarizes the findings of the patients in respect to sources of the diseases.

In 77.4% of the patients, a chronic illness was determined. Cerebrovascular diseases (CVD) were statistically significant in patient group above 75 years (*p* = 0.027).

When chronic diseases were compared according to gender, hypertension (HT) in males and Diabetes Mellitus (DM) and congestive heart failure (CHF) in females were found to be statistically significant (*p* < 0.05).

When accompanying diseases were compared between medical and surgical sources of abdominal pain, HT and liver cirrhosis were significant in medical sources (*p* < 0.05).

Mean values of vital signs of the patients on admission to ED were as follows: systolic blood pressure: 120.0 ± 20.4 mmHg, heart beat: 84.0 ± 13.4 beats/minute, and temperature: 36.6 ± 0.6°C. Blood pressure was found to be significantly higher in females than males (*p* = 0.008).

When medical histories of the patients were investigated, it was determined that 29.8% of the patients have undergone surgery in the past. Malignity was the leading cause for surgery (8%), followed by cholecystectomy (7.1%) and appendectomy (2.9%).

When patients were classified according to duration of abdominal pain (less than 24 hours, more than 24 hours), no statistical significance could be determined between gender, age, and prediagnoses.

For 265 patients (75.9%), a consultation was required. Of the patients admitted, 133 (50.2%) were in 65–74 age group and 132 (49.8%) were in 75 age and above group. When consultations were investigated, 118 (44.5%) patients consulted with internal medicine specialists, 72 (27.2%) with general surgery specialists, 50 (18.9%) with both internal medicine and general surgery specialists, and 25 (9.4%) with other specialists.

The most common diagnosis was biliary and biliary tract diseases (19.6%) followed by nonspecific abdominal pain (11.9%) and abdominal pain related to malignity (9.8%). Subgroups of biliary and biliary tract diseases were choledocholithiasis (11.3%), cholangitis (5.1%), cholecystitis (2.1%), and cholelithiasis (1.2%).

Of the patients, 164 (48.8%) were hospitalized, 131 (39.0%) were discharged from ED, and 41 (12.2%) have died. Of those who were discharged, 107 (81.7%) were discharged with full recovery and 24 (18.3%) rejected treatment and left the ED with written consent.

While majority of the geriatric patients (41.8%) were followed in the observation room of the ED, 24.7% were followed in gastroenterology and 17.6% were followed in general surgery wards.

When LOS of patients were investigated, majority of the patients were determined to stay 0–6 hours. Patients with abdominal pain of medical source had significantly longer LOS (*p* < 0.05).

Of the patients with a prediagnosis of medical source, 2.7% have undergone surgical treatment; of patients with a prediagnosis of surgical source, 26% have undergone medical treatment. We also determined that medical treatment was more likely to be performed when compared to surgical treatment in geriatric patients with abdominal pain.

## 4. Discussion

As the life expectancy of the community increases, number of geriatric patients admitted to EDs increases. Studies on geriatric patients have shown that rate of admission of geriatric patients to EDs varies between 9 and 15% [2, 5, 7, 8]. In our study, percentage of geriatric patients was found to be 21.2%. The reason of the high percentage in our study may be related to characteristics of our hospital as a district hospital that serves as a last step hospital to the city and surroundings.

When complaints of geriatric patients on admission to ED were investigated, Kılıçarslan et al. reported that the most common complaints were chest pain, abdominal pain, shortness of breath, and headache, respectively. Of the patients, 5.7% were admitted due to abdominal pain [9]. Gallenger et al. reported that 5–8% of complaints on admission to EDs were abdominal pain [10]. Additionally, in a study by Fagbohun et al. this rate was reported to be 10% [6]. In concordance with the literature, results of our study revealed that 4.4% of the geriatric patients were admitted due to abdominal pain to our ED.

In the literature, it was well-defined that majority of the patients presented to EDs are females [11–14]. Gardner et al. reported in a study that 60% of the patients were females [15]. Our studies also revealed that majority of the patients were females. Our study also revealed that mean age of women was higher than that of men. Longer life expectancy in women may be the reason for this finding.

The reason for abdominal pain in geriatric patients may originate from both biliary tract infection and pancreatitis. As age progresses, contraction ability of the gallbladder, in response to cholecystokinin enzyme, decreases. Additionally, increased cholesterol and phospholipid content of the bile causes gallbladder stones and increased biliary tract diameter results in biliary diseases [16–18]. In our study, we determined biliary tract diseases in 19.6% and pancreatitis in 4.8% of the patients. Another reason for high rate of biliary tract diseases is related to high transfer rate of the patients for Endoscopic Retrograde Cholangiopancreatography from surrounding cities.

In various studies, the most common complaint accompanying abdominal pain was reported to be nausea/vomiting [4, 11]. In our study, 20.8% of the patients were admitted due to isolated abdominal pain, and nausea and vomiting were the most common accompanying complaints. In surgical source group, gas and stool extraction inability was the most common complaint and this finding was compatible with the results of Staniland et al. [12]. This result is also compatible with the fact that biliary tract diseases are the most common diseases among elderly patients. Its typical clinical presentations are known to be fever, right upper quadrant pain, nausea, and vomiting [19].

In the literature, it was reported that 75–90% of the geriatric patients have a chronical illness [2, 20–22]. Loloğlu et al. reported that the most common chronical illnesses in geriatric patients were as follows: HT (40.8%), coronary artery disease (CAD) (26.6%), and DM (22.4%). In our study, 77.4% of the patients had a chronical illness. The most common illnesses in our study were HT (47.2%), DM (25.7%), and malignity (24%). We also determined that, in 75 years and above group, neurological diseases (Alzheimer's Disease, Parkinson, CVD, etc.) were more common than in 65–74 years group. This finding is reasonable because advanced age is known to be related to higher neurological disease incidence. It is well-described in the literature that HT is a common problem in geriatric patients with a prevalence as high as 60 to 80% [23]. In a study by Salvi et al., it was reported that the most common diagnoses in elderly patients were atrial fibrillation, congestive heart failure, pneumonia, and stroke [24]. In another study, mental status alterations were reported to be present in 1/4 of the elder patients [25]. Additionally, among these diagnoses, neoplasms were determined to have the highest risk for mortality [26].

It is known that the elderly tend to be exposed to infectious diseases, and high body temperature may be a late finding in this patient group [27]. Age is a risk factor for alterations in vital signs [1]. In our study, compatible with the literature, mean body temperature was found to be 36.6 ± 0.6°C. However, we could not find any significance in vital signs when patients were compared according to age groups.

The most common surgical source for abdominal pain in elderly was reported to be biliary and biliary tract diseases [7, 27]. Kauvar et al. reported that acute appendicitis was the third cause of abdominal pain in elderly requiring surgery [28]. In the literature, it is reported that ileus is determined in 10% of the patients with abdominal pain of surgical origin [29–31]. It was also reported that ileus was three times frequent in elderly when compared to younger patients [32]. In our study, the most common reasons for surgery history were malignity, cholecystectomy, and appendectomy. This finding may be related to advanced facilities on malignity surgery in our hospital.

Chronical analgesic use may obscure severity of abdominal pain in elderly [27]. Durukan et al. reported that delayed admittance to hospital in geriatric patients was common (after 97.1 ± 160.8 hours after the onset of the pain). They also reported that signs, symptoms, and findings in these patients might be insignificant, and defense and rigidity might be absent [4]. In our study, tenderness was determined in 79.4%, defense was determined in 20.9%, and rebound was determined in 10.4% of the patients. In concordance with the findings of Pappas et al. [33], abdominal pain in our patients was determined to occur within 24 hours.

The leading lethal condition in patients with abdominal pain is known to be abdominal aortic aneurysm (AAA) rupture [27]. Bengtsson et al. reported that AAA rupture has a prevalence of 2–4% in patients under 50 years. However, prevalence rises up to 10% in patients over 50 years [34]. Another lethal condition in elderly is known to be mesentery ischemia [35]. Despite improvement in diagnostic tests, mortality in mesentery ischemia remains as 60–90% [36, 37]. In our study, 0.3% of our patients had AAA and 3 of our patients had mesentery ischemia. Those with a diagnosis of mesentery ischemia have died after surgery.

It is fact that threshold for consultation in geriatric patients is recommended to be low [7]. Mert et al. reported that 45.4% of the patients with abdominal pain consulted with internal medicine and/or general surgery specialists. It was also reported that the number of consultations was higher in patients above 65 years [1]. In our study a consultation was required for 75.9% of the patients. The higher rate in our study is related to our study design. We only included geriatric patients into our study and consultation rates appeared to be high.

Nonspecific abdominal pain—abdominal pain without a specific origin—is common among young patients. Bavunoğlu et al. reported that nonspecific abdominal pain is seen in less than 15% of geriatric patients [38]. Gün et al. reported in a study that the cause of abdominal pain in 30.6% of the geriatric patients could not be determined and these patients were diagnosed with nonspecific abdominal pain [39]. In our study, incidence of nonspecific abdominal pain was found to be 11.9%. When all ages are considered, nonspecific abdominal pain is the most common complaint in ED; however, in the elderly, biliary tract diseases are known to be more common [40]. Our findings are compatible with the literature.

As age advances, infectious diseases are seen more frequently in elderly patients due to immune system weakening [4]. Our study revealed that the most frequent infectious disease was gastroenteritis followed by urinary tract infections. However, in a study, Saçar et al. reported that urinary tract infection was the most common infection followed by gastroenteritis in geriatric patients [41].

It is well-known that acute appendicitis is more common in young patients when compared to geriatric patients. Numerous studies revealed that the frequency of acute appendicitis in geriatric patients is 5–10% [5, 42, 43]. Durukan et al. reported this rate as 4.5% in their study [4]. In our study, 1.8% of the patients had appendicitis and majority of the patients were involved in 65–74 age group. This finding is compatible with previous findings that incidence of appendicitis decreases by age.

In a study, Türker et al. reported that 33% of the patients with abdominal pain have been discharged with full recovery following medical treatment. Of these patients, 18.4% were discharged for outpatient follow-up, 18.7% were hospitalized in general surgery ward, 9.4% were hospitalized in internal medicine ward, 4.5% were discharged following ED observation, 1.9% left the ED voluntarily, and 0.4% have died [44]. In another study, in 45–64 age group, discharge rate from the ED was reported as 78.6% and hospitalization rate was reported to be 19.6%, while in 65 years and above group, discharge rate from the ED was reported to be 51.7% and hospitalization rate was reported to be 44.5% [1]. In our study, 41.7% of the patients were discharged from the ED, 48.8% were hospitalized, and 12.2% have died. Higher rates of hospitalization and mortality in our study may be related to higher incidence of concomitant chronical illnesses and admittance of patients with bad general condition.

In the literature, it is reported that mortality rates in geriatric patients related to emergency surgery vary between 11% and 37% [45–47]. In our study, mortality rate related to abdominal pain of medical origin was 9.7% and of surgical origin was 20.5%. In the literature there is an ongoing controversy about relationship between gender and mortality. Ağalar et al. reported that mortality rate in males is high when compared to females [48]. However, Reis et al. reported that gender did not have any influence on mortality rates [49]. In our study, mortality rate was found to be higher in both 75 years and above group and female gender.

In our study, the most common ward where the patients were hospitalized was found to be internal medicine, particularly gastroenterology ward. It was previously reported that hospitalization rates of patients with abdominal pain vary between 18.9% and 63.2% [4, 5, 43]. Pappas et al. reported that patients above 65 years were more likely to be hospitalized in internal medicine wards [42]. Mert et al. reported that general surgery was the most commonly preferred ward for patients with abdominal pain [1].

In a report by Chan et al., it was stated that 1.4–2.9% of the patients admitted to EDs leave the ED without being seen by a physician due to prolonged LOS in the ED [50]. This rate was reported to be 1% by Serinken et al. [51]. In our study, 68% of the patients had to wait more than 6 hours in the ED. Of the patients, 7.1% refused treatment and left the ED. While refusal rate in patients with abdominal pain of medical origin was found to be 8.5%, it was 2.6% in surgical origin group. This may be a result of prolonged LOS and delay in diagnosis of patients with abdominal pain of medical origin.

In the literature, medical treatment is the most common treatment method in geriatric patients with abdominal pain [42, 52, 53]. On the contrary, Pappas et al. could not find any difference between medical and surgical procedures in patients under 65 and above 65 years [33]. In our study, we determined that surgical treatment was performed to 17.6% of our patients. In 65–74 years group, surgery rate was 14.2%, and in 75 years and above group, surgery rate was 21.2%.

## 5. Conclusion

Results of our study revealed that the most common cause for abdominal pain in geriatric patients is biliary tract disorders. We also determined that majority of the patients with abdominal pain have a concomitant chronical illness. The most common complaints accompanying abdominal pain are nausea/vomiting and gas extraction inability. Mortality rate in geriatric patients with abdominal pain is 12.2% and higher in males. As the age advances, both rates of surgical procedures and mortality rate increase.

## Figures and Tables

**Table 1:** Comparison of patient characteristics according to age groups.

**Characteristics **	**Group I: 65–74 years (** **n** **, %)**		**Group II: above 75 years (** **n** **, %)**	
**Total patient number**	176	52.4	160	47.6

**Gender**				
Male	94	58	68	42
Female	82	47.1	92	52.9

**Vital signs**				
Blood pressure (mmHg)	110 ± 19.09		120 ± 21.9	
Heart rate (beats/min)	80 ± 13.8		86 ± 12.8	
Fever	36.6 ± 0.7		36.6 ± 0.5	

**Complaints accompanying abdominal pain**				
Nausea	88	50,3	78	48.8
Vomiting	72	41,1	62	38.8
Loss of appetite	22	12.6	13	8.1
Constipation	28	16	17	10,6
Intestinal gas extraction inability	25	14,3	13	8,1
Diarrhea	19	10,9	18	11.3
Dysuria	19	10,9	16	9.4
Jaundice	19	10,9	19	11.9

**Length of ED stay**				
0–6 hours	74	42	68	42.5
6–12 hours	49	27.9	61	38.1
12–24 hours	24	13.7	11	6.9
Over 24 hours	29	16.4	20	12.5

**Duration of abdominal pain**				
0–24 hours	35	20	140	80
Over 24 hours	33	20.6	127	79.4

**Length of stay in the hospital**				
1 day	76	43.2	64	40
1–4 days	20	11.4	25	15.6
4–10 days	38	21.6	33	20.6
More than 10 days	42	23.8	38	23.8

**Choice of treatment**				
Medical	151	85.8	126	78.8
Surgical	25	14.2	34	21.2

**Consultation**				
Internal medicine	60	45.1	58	43.5
General surgery	35	26.3	37	28.2
Internal medicine and general surgery	22	16.5	28	21.4
Other consultations	16	12	9	6.9

**Outcomes **				
Recovery	73	41,5	58	36,3
Vegetative stage	88	50	76	47.5
Exitus	15	8.5	26	16.2

**Table 2:** Comparison of groups according to diagnoses.

**Characteristics **	**Internal prediagnoses (** **n** **, %)**		**Surgical prediagnoses (** **n** **, %)**		**p** ** value**
**Total patient number**	258	76.8	78	23.2	

**Gender**					
Male	121	46,9	41	52,6	
Female	137	53,1	37	47,4	

**Vital signs**					
Blood pressure (mmHg)	120 ± 19.1		120 ± 24,1		
Heart rate (beats/min)	84 ± 12.7		84 ± 15.7		
Fever	36.6 ± 0.6		36.7 ± 0.5		

**Complaints accompanying abdominal pain**					
Nausea	123	47.7	43	55.1	
Vomiting	96	37.7	38	48.7	
Loss of appetite	31	12	4	5.1	
Constipation	31	12	14	17.9	
Inability to intestinal gas extraction	14	5,4	24	30.4	0,001
Diarrhea	34	13.2	3	3.8	0,021
Dysuria	28	10.9	7	9	
Jaundice	36	14	2	2.6	0,005

**Length of ED stay**					
0–6 hours	117	45,3	24	30,8	0,002
6–12 hours	73	28,3	38	48,7	
12–24 hours	25	9,7	10	12,8	
Over 24 hours	43	16.7	6	7,7	

**Duration of abdominal pain**					
0–24 hours	55	21,3	13	16,7	
Over 24 hours	203	78,7	65	83,3	

**Length of stay in the hospital**					
1 day	126	48.8	13	16,7	0,001
1–4 days	30	11.6	15	19,2	
4–10 days	48	18.6	23	29,5	
More than 10 days	54	21	27	34,6	

**Choice of treatment**					
Medical	251	97,3	26	33,3	0,001
Surgical	7	2,7	52	66,7	

**Consultation**					
Internal medicine	114	59,7	4	5,4	0,001
General surgery	22	11,5	50	67,6	
Internal medicine and general surgery	33	17,3	3	4,1	
Other consultations	22	11,5	17	23	

**Outcomes **					
Recovery	120	46,5	11	14,1	0,001
Vegetative stage	113	43,8	51	65,4	
Exitus	25	9,7	16	20,5	

## Data Availability

The data used to support the findings of this study are available from the corresponding author upon request.
